# Identifying priority wetland sites in the East Asian-Australasian Flyway for migratory bird conservation

**DOI:** 10.1038/s41598-025-31727-2

**Published:** 2025-12-11

**Authors:** Mike Crosby, Shelby Q. W. Wee, Ding Li Yong, Gary Allport, Sayam U. Chowdhury, Gan Xiaojing, Ward Hagemeijer, Arne E. Jensen, Duncan A. I. Lang, Cynthia Layusa, Yoon Kyung Lee, Taej Mundkur, Heejin Oh, Shi Jianbin, Terry Townshend, Doug Watkins, Qing Zeng, Lenke Balint, Stefano Barchiesi, Radhika Bhargava Gajre, William Fairburn, Daniel A. Friess, Tom Lambert, Hui Koon Lim, Karen G. C. Ochavo, Evelyn Pina-Covarrubias, Hao Tang, Kelvin S.-H. Peh

**Affiliations:** 1 BirdLife International, Cambridge, UK; 2 BirdLife International Asia Division, Singapore, Singapore; 3 Conservation Science Group, Department of Zoology, University of Cambridge, Cambridge, UK; 4 Paulson Institute, Beijing, China; 5 Wetlands International, Wageningen, The Netherlands; 6 Wild Bird Club of the Philippines, Pasay City, Philippines; 7 Asian Development Bank, Manila, Philippines; 8 East Asian-Australasian Flyway Partnership, Incheon, Republic of Korea; 9 Beijing Normal University, Beijing, China; 10 Centre for Nature-based Climate Solutions, National University of Singapore, Singapore, Singapore; 11 Department of Earth and Environmental Sciences, Tulane University, New Orleans, USA; 12 School of Biological Sciences, University of Southampton, Southampton, UK

**Keywords:** Asian Waterbird Census, Key biodiversity areas, IUCN red list, Ramsar, Convention on wetlands, Waterbird conservation, Wetland conservation, Environmental sciences, Biodiversity, Conservation biology, Tropical ecology, Wetlands ecology

## Abstract

The East Asian-Australasian Flyway (EAAF) is widely recognised to be the most threatened of the eight flyways in the world, with wetlands rapidly lost due to land cover change, unsustainable use, and the wider impacts of climate change. The recently established EAAF Regional Flyway Initiative (RFI) aims to bring a set of priority wetlands in the EAAF under improved protection, management, and restoration in 10 Asian countries, while mobilising resources for sustainable agriculture, aquaculture, ecotourism, and other livelihoods for local communities. A major step in the development of this initiative is the identification of priority wetland sites through the application of international criteria, based on modern waterbird count data collated from wetland sites across Asia. Through existing analyses and expert consultations, we short-listed a minimum of 270 internationally important wetlands as candidate localities for further assessment. Count data of EAAF waterbird species was then assessed against international criteria aligned with the Convention on Wetlands (Ramsar Convention), the EAAF Partnership’s Flyway Site Network and Important Bird and Biodiversity Areas for each site to iteratively identify a subset of priority sites, drawing on newly available species population thresholds. Each site was scored and ranked using a metric (Prioritisation Criterion 1) calculated from the proportions of every occurring EAAF species against published population thresholds. We identified a total of 147 wetland sites of high conservation priority across the 10 countries, both freshwater and coastal. At least 34 threatened species, including significant proportions of their global populations are represented in this set of 147 sites. To ensure that conservation opportunities are maximised for species and ecosystem services, there is a need to ensure that selected sites and landscapes are reconciled with the conservation and development priorities of each country, ecological connectivity and to evaluate priority sites for their ecosystem services.

**Supplementary Information:**

The online version contains supplementary material available at 10.1038/s41598-025-31727-2.

## Introduction

Long-distance migratory waterbirds rely on a network of interconnected and highly productive wetlands for breeding, resting, and refuelling during their annual journeys. These migrations are vital for the survival of many species. Among the major migratory pathways in the world, the East Asian-Australasian Flyway (EAAF) stands out as one of the most significant and biologically diverse. The EAAF, spanning from the Arctic tundra of central and eastern Russian Federation and Alaska (United States), through East Asia and Southeast Asia to Australasia, represents a vital pathway for over 50 million migratory waterbirds from more than 270 different populations. Notably, this flyway supports at least 34 globally threatened migratory species, making it the most threatened among the eight globally recognised flyways^1,2^. The EAAF’s importance is highlighted by its role in supporting long-distance migratory waterbirds^3,4^. Therefore, wetland habitats in EAAF is essential to safeguard the future of these migratory species.

Yet, bird migrations face substantial threats due to the accelerated loss and degradation of wetland habitats^5,6^. This can be attributed to rapid drainage and conversion of wetlands to agricultural, industrial, and urban uses, which have led to the alarming decline of these ecosystems^5,7^. For example, an estimated 1,794.8 km^2^ (29%) of coastal wetlands in the Yellow Sea (bordered by People’s Republic of China [PRC] and Korean Peninsula) and Bohai Sea (off the northern coast of PRC) were lost to development between 2000 and 2015 due to conversion for aquaculture and salt pans^8–10^. Historical data suggests that up to 65% of tidal flats in the region have also been lost there over the past five decades^11^. Thus, the migratory waterbirds that they support face a wide range of pressures, including loss of secure roosting sites, illegal hunting, pollution, and the wider impacts of climate change^12–14^. Consequently, an increasing number of migratory waterbird species within the EAAF are in rapid decline^15^, many which have been classified under the IUCN Red List of Threatened Species^16,17^ as globally threatened. Some of these species have become flagships for the EAAF and the focus for monitoring and conservation actions, including the Spoon-billed Sandpiper *Calidris pygmaea*^18^, Black-faced Spoonbill *Platalea minor*^19^ and several threatened crane^20^, geese^21^ and duck species^22^.

The loss and degradation of wetland sites not only threatens biodiversity but also significantly impacts human communities and economies reliant on these ecosystems. Fisheries and vital ecosystem services (e.g., nature-based tourism, seaweed cultivation) are in danger of collapse and ecological disasters are increasing, with concomitant implications for human communities. For example, wetland loss as a result of conversion to farmland in the Sanjiang Plain in northern PRC (which includes the Sanjiang National Nature Reserve) has reduced ecosystem service value by approximately $57.46 billion over the past six decades^23^. Therefore, the stakes are very high, as wetland loss could result in financial setbacks for the agricultural and fisheries sectors and potential economic damage to coastal cities, towns, and agricultural lands^24–26^.

Urgent long-term and large-scale action is required to address this ecological and socio-economic crisis, thereby securing not only the integrity of wetland sites and landscapes in the EAAF, but also the wellbeing of local human populations and the adaptation to climate change. The Asian Development Bank (ADB), BirdLife International and the East Asian-Australasian Flyway Partnership (EAAFP) launched the Regional Flyway Initiative (RFI) in October 2021^27^, a regional initiative aimed at strengthening the management of wetlands of high conservation priority across 10 Asian countries. A major goal of this initiative is to enhance the conditions of a set of important wetlands that supports migratory connectivity, contributing to the protection of migratory waterbirds while promoting transboundary cooperation. This initiative is also expected to yield measurable co-benefits for local communities, including ecosystem services, economic development, green infrastructure, and climate change mitigation and adaptation^28^.

A major challenge in biodiversity conservation and natural resources management is the efficient allocation of scarce resources to address complex environmental problems^29^. Often a process of ‘conservation triage’ is necessary to ensure that resources are allocated to where they can make the largest impact, for instance the conservation of landscapes with high species richness or threatened species, potentially by accounting for the costs and benefits, and the likelihood of success^29–31^. Conservation prioritisation therefore forms a critical part of conservation decision-making by mobilising resources to landscapes, species, and ecosystems that are most threatened. However, prioritising sites/landscapes and species for conservation interventions is data-hungry and can be challenging if data used in the prioritisation process is limited or is collected in a non-standardised way^32^. Moreover, information used for prioritisation such as conservation costs and anthropogenic threats may be highly uncertain or difficult to obtain^33^. Prioritising sites and landscapes for the conservation of migratory species can also be challenging at large spatial scales because count or abundance-based datasets on migratory species tend to be scarce or are unevenly collected (variable sampling effort) because of the different methodologies used to survey species.

Numerous approaches to prioritising sites and landscapes for biodiversity conservation exist and can range from simple counts of species richness at a site to complex mathematical and optimisation approaches based on the representation of sets of species and biodiversity/ecological features in a set of sites^31,34^, and how sites can complement others under the framework of systematic conservation planning^30^. An important consideration in conservation prioritisation is the principle of irreplaceability. Irreplaceability can be defined as the potential and relative contribution of a site to a wider conservation goal, and considers the extent to which options for a representative network of sites are lost, if one particular site within the network is lost^35^. Therefore, sites and landscapes can be regarded as highly irreplaceable if they are considered to represent a high proportion of the conservation features (i.e., species, biomes) present in an area considered.

This paper aims to outline the quantitative criteria and methodology used to identify the priority wetland sites earmarked for RFI intervention. The process entails compiling an extensive candidate list of wetland sites of perceived high importance across 10 Asian countries, followed by systematic scientific assessments to pinpoint high-priority sites. The identification of these high-priority sites is standardised by rigorous scientific principles (e.g., irreplaceability) and evidence-based criteria, ensuring credibility and effectiveness. This would serve as specific guidance for assessing site conservation opportunities. Our study builds on the work of Jaensch^36^ who identified a set of important wetland sites in the EAAF by compiling waterbird count data across the flyway and ranking them based on their contribution to internationally important waterbird populations, with the most effective method being the percentage contribution of these populations supported by each site. These priority sites may now be outdated or have changed, highlighting the need for updated site information to ensure effective conservation efforts. Our study also addresses the previous lack of detailed site boundary maps, a gap noted by Jaensch^36^ due to limited resources. By creating these maps, we can improve the documentation of ecosystem services provided by the sites and develop site-specific conservation intervention plans.

Recognising that the ecological and conservation value of wetlands can vary according to their type, protection status, and human influence, we focus on these attributes to determine the characteristics of the selected priority wetland sites. Specifically, we analysed the priority sites for associations with wetland types (coastal versus inland), protection status (protected versus unprotected), and levels of human development; these associations could reveal critical insights into the vulnerabilities and resilience of different wetland categories. Such knowledge is essential for formulating targeted conservation interventions, policies and site management^37^. The implications and lessons gleaned from our analyses can offer broad insights and guidance for the development of other regional initiatives and conceptual frameworks for the conservation of migratory species and wetlands at the regional scale, for instance frameworks in the Americas and Europe/Africa.

## Methods

**Fig. 1:**
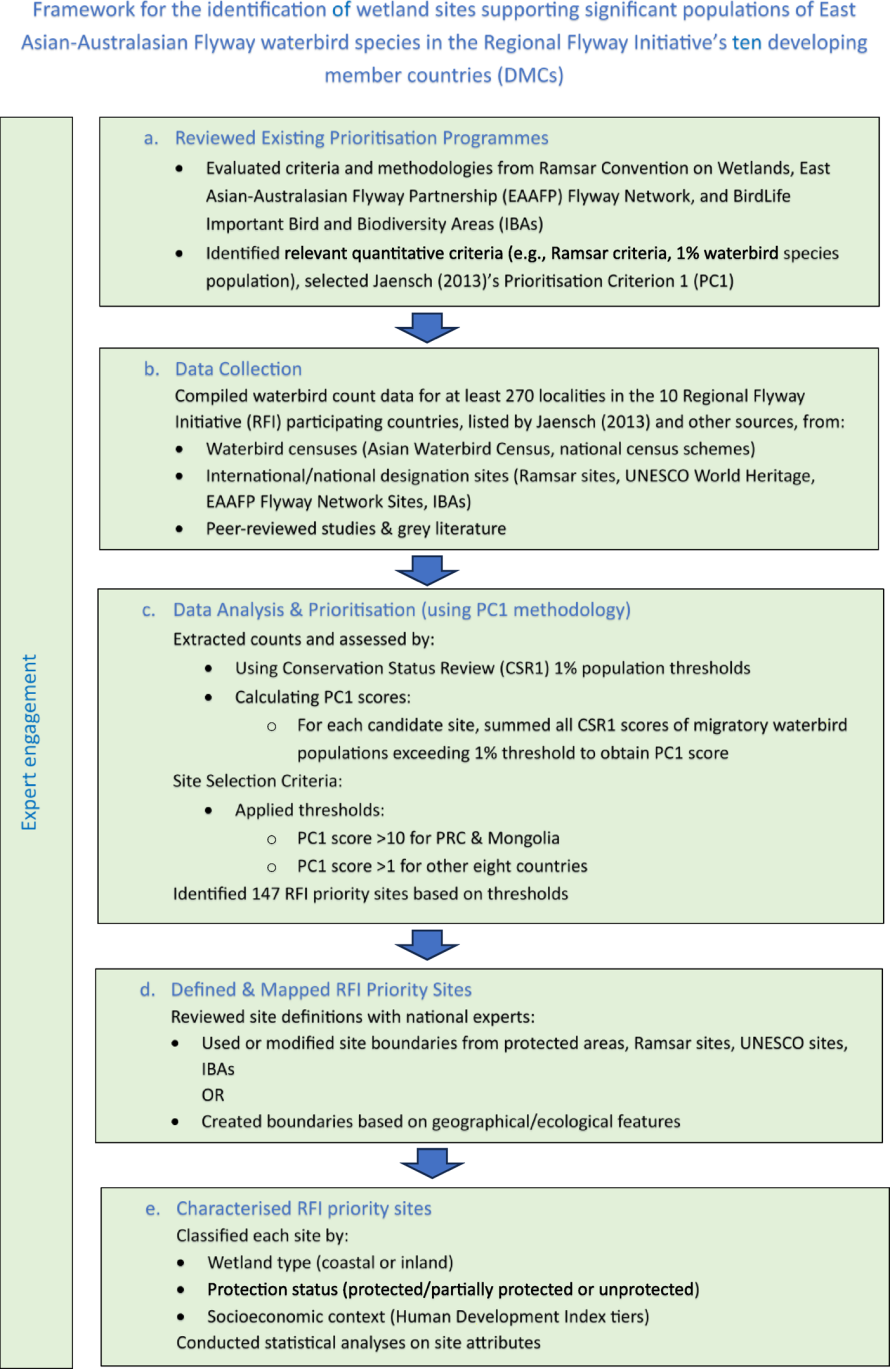
Regional Flyway Initiative (RFI) site selection framework for the East Asian-Australasian Flyway (EAAF): key steps in the site prioritisation process.

This study outlines the criteria and methodology used to identify and rank a set of high priority wetland sites in the 10 RFI countries in the EAAF. The main stages in this process were: (a) a review of existing site prioritisation programmes, to identify the most appropriate criteria and methodology to use in the current study; (b) collection of data on migratory waterbirds and wetland sites; (c) preparation and analysis of waterbird count data, using the Prioritisation Criterion 1 (PC1) methodology chosen during the review of prioritisation schemes and the new Conservation Status Review (CSR1) 1% population thresholds; (d) definition of the RFI priority sites and their boundaries; and (e) characterising the RFI priority sites (Fig. 1).

### Review of existing site prioritisation frameworks

#### Evaluating criteria

To prioritise wetlands of high conservation importance, we evaluated internationally accepted criteria adopted by three long established government-endorsed frameworks for biodiversity conservation (Table 1). These are:

**Table 1:** Internationally recognised designations of wetland sites in the regional flyway initiative (RFI) countries from the Ramsar convention on wetlands of international Importance, the East Asian-Australasian flyway partnership (EAAFP) flyway site Network, and the Birdlife International Partnership’s important bird and biodiversity areas (IBAs) or key biodiversity area (KBA) programme (as of 31 March 2023). These wetland sites were considered as candidates during the prioritisation process, but not all of them met the necessary criteria to be designated as RFI priority sites (see Table 3).

RFI country	Government endorsed programme		
	Ramsar sites	EAAFP network sites	Wetland IBA/KBA
Bangladesh	2	6	9
Cambodia	5	1	35
PRC	82	20	221
Indonesia	7	2	71
Lao PDR	2	N/A	15
Malaysia	7	1	16
Mongolia	11	11	39
Philippines	8	4	17
Thailand	15	3	17
Viet Nam	9	1	25
Total	148	49	465

1. The *Ramsar Convention on Wetlands of International Importance*^38,39^ which provides a framework for international cooperation for the conservation and wise use of wetlands. Contracting Parties of the Ramsar Convention designate suitable wetlands that have met its criteria^40^ for inclusion in the list of Wetlands of International Importance, commonly known as Ramsar sites. All 10 RFI countries are Contracting Parties to the Convention and had designated a total of 148 Ramsar sites (as of 12 September 2023).
2. The *East Asian-Australasian Flyway Partnership Flyway Site Network*^41^ which is a semi-formal network of wetland sites, typically nominated by government partners after having been identified for their international importance to migratory waterbirds in the EAAF. The EAAFP is a voluntary initiative established to protect migratory waterbirds, their habitats, and the livelihoods of people dependent upon wetlands, and consists of 41 Partner organisations including 18 national governments. The governments of nine of the RFI participating countries are EAAFP Partners and they have collectively designated a total of 49 Flyway Network sites (as of 12 September 2023)^41^.
3. The BirdLife International Partnership’s *Important Bird and Biodiversity Areas (IBAs) Programme*^42,43^ which is an global initiative that has identified and documented more than 13,000 IBAs worldwide, which are sites of international significance for the conservation of birds and other biodiversity based on a measurable set of criteria. IBAs are typically also Key Biodiversity Areas (KBAs)^44^. In total, 465 wetland IBAs and KBAs have been identified to date in the 10 RFI participating countries (as of 12 September 2023)^45,46^.

#### Identifying relevant quantitative criteria

A set of nine quantitative criteria has been developed under the Ramsar Convention to identify Wetlands of International Importance, including the four that are relevant to waterbirds (for details see Table 2). Criteria 2, 5 and 6 are used by the EAAFP to identify Flyway Network Sites for migratory waterbirds in the EAAF, and Criteria 2 and 6 have been adopted by BirdLife International to identify IBAs for globally threatened and congregatory waterbirds worldwide.

**Table 2:** The criteria for identifying wetlands of international importance (Ramsar Sites) that are relevant to waterbirds (four of the nine Ramsar criteria; see Ramsar^38,40^.

The Ramsar Sites Criteria
Criteria based on species and ecological communities
Criterion 2: A wetland should be considered internationally important if it supports Vulnerable, Endangered, or Critically Endangered species or threatened ecological communities
Criterion 4: A wetland should be considered internationally important if it supports plant and/or animal species at a critical stage in their life cycles or provides refuge during adverse conditions
Criteria based on waterbirds
Criterion 5: A wetland should be considered internationally important if it regularly supports 20,000 or more waterbirds
Criterion 6: A wetland should be considered internationally important if it regularly supports 1% of individuals in a population of one species or subspecies of waterbird

The 1% population thresholds used under Ramsar Criterion 6 are available from Wetlands International through the Waterbirds Populations Portal^47^ (WPP), an online, open-access database which provides current and historic population estimates and 1% population thresholds and population boundary maps for definable biogeographic populations of all waterbird species worldwide^48^. We used a new set of 1% population thresholds for migratory waterbirds published in the first East Asian - Australasian Flyway Conservation Status Review (CSR1)^1,48^. These thresholds are now the official list for the application of the Ramsar site criteria in the EAAF (for migratory waterbirds only, as the CSR1 review did not cover non-migratory populations) and are the most authoritative source of information on waterbird populations to use in the RFI site prioritisations.

Jaensch^36^ conducted a flyway-wide assessment of wetland sites and their relative importance to migratory waterbirds at the regional scale to help guide the designation of new sites in the EAAFP’s Flyway Sites Network. This study compiled a large dataset of waterbird count data from localities throughout the EAAF (a substantial proportion collected through the annual Asian Waterbird Census coordinated regionally by Wetlands International) and identified over 1,000 potential Flyway Network sites, including 467 sites in the 10 RFI participating countries. In the following section, we outline the site prioritisation criteria tested by Jaensch^36^, to determine which of them was the most effective to select and rank potential EAAFP’s Flyway Sites:

- Prioritisation Criterion 1 (PC1): Derived from the internationally important waterbird populations (defined as those that regularly support 1% or more of the individuals in a population of a waterbird species and meet Ramsar Criterion 6) which have been recorded at the site; the percentage of each internationally important population was calculated and the resulting percentages summed to give the PC1 score.
- Prioritisation Criterion 2 (PC2): The number of internationally important waterbird populations (defined as those that regularly support 1% or more of the individuals in a population of a waterbird species and meet Ramsar Criterion 6) which have been recorded at the site.
- Prioritisation Criterion 3 (PC3): The number of globally threatened populations (in IUCN Red List categories Critically Endangered, Endangered or Vulnerable and meeting Ramsar Criterion 2) which have been recorded at the site.

Jaensch^36^ concluded that Prioritisation Criterion 1 (PC1) was the most useful metric as a relative measure of the contribution of each site to the conservation of migratory waterbirds in the flyway, as it resulted in both a list of priority sites and a ranking of these sites according to their importance for migratory waterbirds. We therefore decided to use the PC1 methodology in the current study. Xia et al.^49^ prioritised coastal wetlands according to their importance to migratory waterbirds using a similar prioritisation methodology to Jaensch’s^36^ PC1 (but referred to by Xia et al.^49^ as the “irreplaceability index”), represented with the formula:

$$I = \sum\limits_{i = 1}^S {{n_i}/N} \times 100,$$

where *I* demotes PC1 score, *n*_*i*_ denotes the population of *i*th species of waterbirds at the survey site, *N* denotes the population of *i*th species globally or throughout the flyway, and s denotes the number of species at the survey points.

### Collection of data on migratory waterbirds and wetland sites

We compiled data on the numbers of migratory waterbirds counted at wetland sites in the 10 RFI countries (for details see Supplementary Tables 1 and 2). The main sources of data are outlined below.

#### Waterbird count data compiled for site prioritisation analyses

The 467 bird count localities listed by Jaensch^36^ for the 10 RFI participating countries formed the foundation for the RFI sites prioritisation analysis and provided a large (but relatively dated) database of waterbird count records. Additional data on the definition and locations of sites and waterbird abundances (maximum counts) was compiled from flyway-wide analyses of EAAF wetland sites by MacKinnon et al.^24^ and important shorebird sites by Conklin et al.^50^, and the analyses of priority wetlands in the PRC by Paulson Institute^51^, Xia et al.^49^, Zhang et al.^52^ and Duan et al.^10,53^.

#### Internationally and nationally designated sites

Data on Ramsar sites, UNESCO World Heritage Sites^54^, EAAFP Flyway Network sites and IBAs was compiled, including boundary maps, site information sheets and reports with waterbird count data. The boundary maps of protected areas, where available, were downloaded from the World Database on Protected Areas^55^ (WDPA).

#### Asian Waterbird Census (AWC)

The AWC^56^ gathers information annually on waterbird populations at wetlands in the region during the non-breeding period of most species (January, including counts from December to February), as a basis for the evaluation of sites and monitoring of populations^57^. Together with the historical AWC data published from 1987 to 2015, this formed the largest source of data for our analyses^57,58^.

#### National and flyway-wide waterbird monitoring schemes

Data on internationally important counts of migratory waterbirds at the site level was obtained from diverse sources. In the PRC, the China Coastal Waterbird Census^59,60^ provided the main source of count data for species at the site level. Additionally, site-level count data was also compiled based for non-breeding waterbirds in the Central and Lower Yangtze river basin^61,62^, Northeast China^63,64^ and in the Yellow Sea-Bohai based on large scale counts^65^.

Data on sites across the EAAF was compiled from field projects under the Global Flyway Network^66^ (e.g., Hassell et al.^67^, Piersma et al.^68^, the International Black-faced Spoonbill Census^69^, the Spoon-billed Sandpiper Winter Census^70^, and other waterbird survey projects funded by the EAAFP and conservation organisations such as BirdLife International, Wildlife Conservation Society (Cambodia and Lao People’s Democratic Republic [PDR]), Manfred Stiftung/Eksai foundations (Indonesia) and IUCN (Lao PDR)).

#### Peer-reviewed scientific papers and survey project reports

A literature search was conducted to identify peer-reviewed articles that held count data on migratory waterbirds and wetland sites in the EAAF. This covered every issue of six relevant bulletins and journals from 2010 to 2021; they include *Wader Study*, *Stilt*, *Bird Conservation International*, *Forktail* (subsequently *‘Journal of Asian Ornithology*’), *BirdingAsia* and *Kukila*.

#### Expert opinion and other sources of data

We consulted national experts in the 10 RFI countries (see Acknowledgements) who provided information on the relative importance of many of the candidate priority sites and advised on other sources of grey literature on wetland sites and waterbirds. We also consulted other sources of data, notably waterbird counts at potentially important sites downloaded from the eBird website^71^, which are validated by national experts, several of whom are authors of this paper (TM, AJ).

### Preparation and analysis of waterbird count data

We designed a database to organise records of the waterbird count data for a minimum of 270 candidate sites identified from the previous step and analysed it using the PC1 methodology (see Supplementary Tables 1 and Dataset). Records of waterbird count data was extracted from the data sources outlined above and compiled into the database (see Supplementary Dataset). For each species detected, we recorded the locality where the count was made and the source of the count data, the number of individuals counted, and the count date. The counts on each row were divided by the CSR1 threshold to give the CSR1 score. The CSR1 scores over 1.0 were then summed to calculate the overall PC1 metric for each site. If more than one count exceeded 1.0 for a species in a site, the most recent of the counts was used for the PC1 calculation.

The main source of waterbird count data in most countries was the AWC, mostly comprising post-2015 records and often a series of annual surveys that allowed us to calculate average waterbird counts for the priority sites. A much larger and more variable dataset was compiled for PRC from published papers and reports, where we used the most recent available data for the PC1 calculations, almost all post-2005 and mostly post-2015. Datasets collected before 2005 were used only in exceptional cases, notably for the Dongsha Shoal in Jiangsu Province, PRC^72^, which has not been surveyed in recent decades but retains very extensive intertidal wetlands. For candidate RFI sites lacking recent waterbird counts, notably in the Yellow River Basin and northeast PRC, experts were consulted to investigate whether the condition of the wetland habitats remains stable (i.e., not degraded) and are therefore likely to retain important waterbird populations. Only a small number of studies in PRC have published multi-year surveys that enabled us to calculate average waterbird counts, and in most cases, we had to use maximum counts from a single year.

The PC1 metric for wetland sites in PRC and Mongolia tended to be much higher than sites in Southeast Asia, in part because the wetlands in these two countries are generally extensive and support large numbers of staging (rather than wintering) migratory waterbirds, but also because they support a much higher diversity of migratory waterbird species. For example, many species of Anatidae (swans, geese and ducks) and cranes occur in the northern part of the flyway, but few of them reach Southeast Asia. To take account of these differences, the threshold PC1 score was set higher at 10.0 for PRC and Mongolia, but at 1.0 for the other eight RFI countries. We chose a threshold of 10.0 based upon a review by waterbird experts of our China and Mongolia site data. The consensus was that waterbird congregations in these two countries will be typically and inevitably larger, because of the (ecological) nature of wetlands there as staging sites for EAAF waterbirds (versus wetland sites in tropical Asia, where bird congregations are expectedly smaller, and more scattered). This threshold was selected through discussion and agreement among authors, experts, and applied on all sites in China and Mongolia to ensure consistency and relevance for our analysis. There were, however, exceptions where three Mongolian sites were identified by the national experts as priority despite having a score < 10.0 (Tolbo Lake = 9.4; Terkhiin Tsagaan Lake = 6.0; and Buir Lake = 8.0).

### Definition of the RFI priority sites and their boundaries

A candidate list of potential RFI priority sites was developed during the data-gathering phase of the study, based upon information relevant to the definition, naming and mapping of sites extracted from the references outlined above. We worked with national experts in the 10 RFI countries to define and delineate sites that are ecologically meaningful and have the potential to be managed as conservation units for migratory waterbirds, following guidelines developed by BirdLife’s IBA Programme, and the AWC Guidelines^73^ on count site mapping developed by Wetlands International. Many waterbird count localities have been clustered together within the RFI sites, and the waterbird count data from these localities is included as part of the site documentation.

We obtained or created boundary maps for the RFI priority sites from several sources. Where the site boundaries were based upon a formal protected area, the maps were generally available to download from the World Database on Protected Areas^55^ (WDPA). Some boundaries were taken or adapted from the Ramsar list of Wetlands of International Importance, the databases of UNESCO World Heritage Sites^54^, EAAFP Flyway Network sites and IBAs, or were provided by national governments. Where no existing map of a site was available, we delineated boundaries based on expert opinion, and geographical and ecological features. The definition, naming and mapping of the sites has provided the foundation for the later stages of the RFI, including ecosystem services assessments and the identification of potential site interventions to benefit waterbirds and local communities.

### Characterising the RFI priority sites

We classified all RFI prioritised sites broadly according to their wetland types (either coastal or inland), including artificial wetlands such as salt pans and fishponds, which are present in many of the priority sites, and typically provide important feeding and roosting habitats for migratory waterbirds. Coastal wetlands are defined as those found along coastlines and estuaries, characterised by varying salinities due to mixing of seawater and fresh water, creating challenging conditions for most plants; they encompass tidal flats, a vital feeding habitat for a suite of specialised waterbirds, salt marshes and mangroves^74^. Inland wetlands are diverse, occurring on floodplains, depressions, and low-lying areas, with varying water presence; they encompass lakes, rivers, marshes, swamps, and wooded swamps dominated by different types of vegetation^74^.

We classified each priority site as either (1) a protected or partially protected area, or (2) an unprotected area, based on available information about the site and expert knowledge. We considered a protected area as a distinctly defined geographic area that is identified, designated, and formally administered, either through legal methods or other effective approaches (e.g., site-based programmes), to ensure the long-term preservation of nature, along with its linked ecosystem benefits and cultural values (following IUCN definition^75^. Comparing the proportions of the two wetland types in protected and unprotected areas can identify protection gaps, evaluate effectiveness, and highlight areas that need more conservation efforts.

Lastly, we assigned each priority site a human development index (HDI), which is derived for their country based on a diverse range of socioeconomic indicators, such as life expectancy, literacy rate, access to electricity in rural areas, GDP per capita, trade activity, homicide rate, multidimensional poverty index, income inequality, internet accessibility, and numerous others^76^. These indicators are synthesised into a metric ranging from 0 to 1.0, with 1.0 representing the highest level of human development. The HDI is then categorized into four tiers: very high human development (0.800-1.000), high human development (0.700-0.799), medium human development (0.550–0.699), and low human development (below 0.550). Countries with low human development tend to have unstable governments, widespread poverty, limited access to health care, poor education, low income, and low life expectancies.

We used Chi-square goodness of fit test to analyse if the selected priority sites are associated with a particular wetland type, or HDI tier. We used 2 × 2 contingency table Chi-square test to compare proportions of the two wetland types distributed between the protected/partially protected and unprotected categories.

All analyses were performed using R (version 4.4.3)^77^.

## Results

### Outcomes of RFI priority site identification

**Fig. 2:**
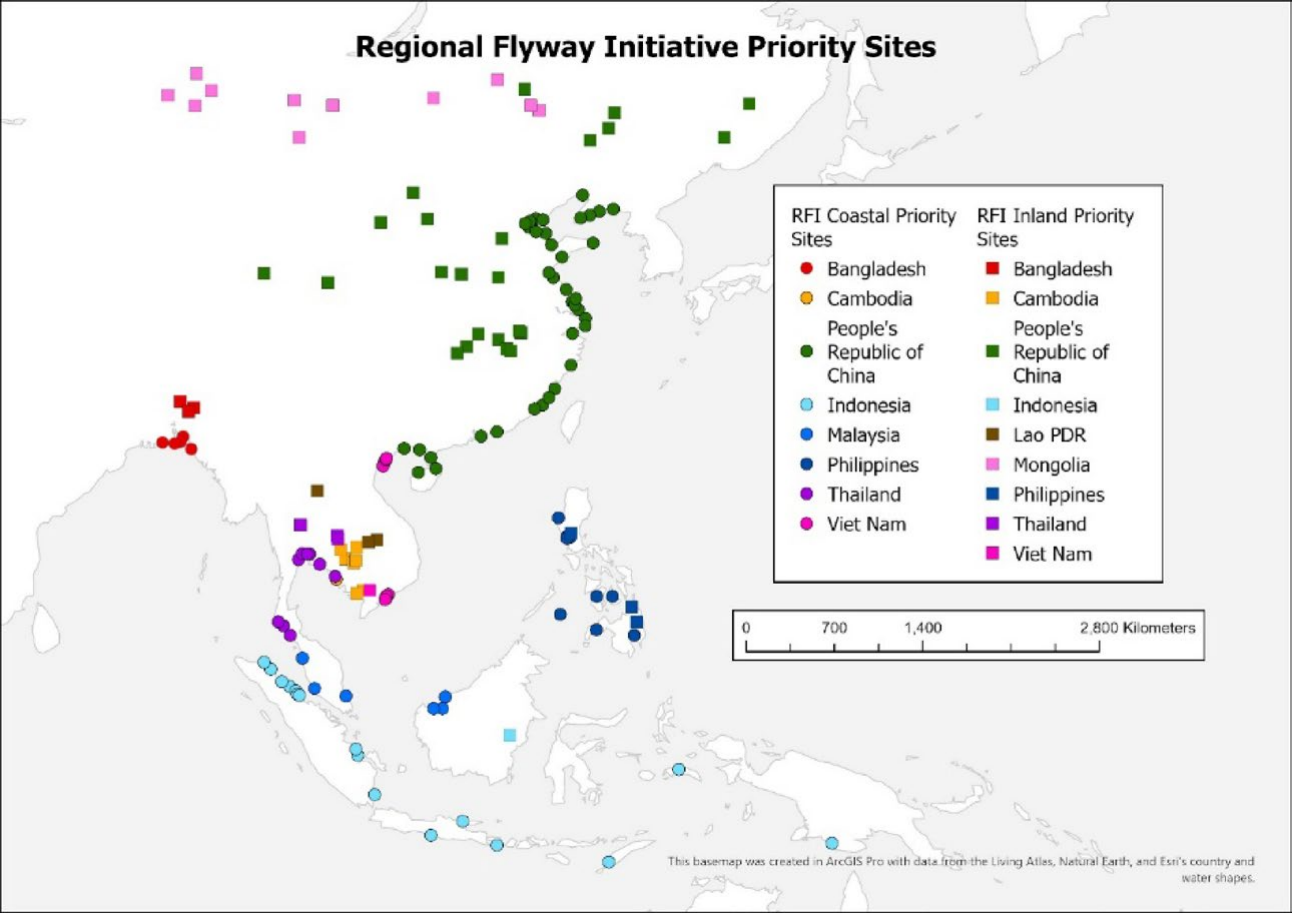
Regional Flyway Initiative (RFI) priority sites (see Table 4 for the human development index for each country).

A total of 147 RFI priority sites was shortlisted from a minimum of 270 wetland sites, which were initially selected from at least 467 candidate sites across 10 countries. (Fig. 2; Tables 1 and 3; Supplementary Tables 2 and 3). The priority sites range from three in Lao PDR to 60 in the PRC, and all 10 countries have various opportunities to develop site-based investments. They include 91 wetland sites located on or near the coast (sites dominated by mangroves and other intertidal wetlands), and 56 inland (mostly freshwater) wetlands along major river basins (Table 4). Table 3 summarises the data gathered on species detected in large congregations across priority sites in the 10 RFI countries.

**Table 3:** Wetland sites in each of the 10 participating countries, including the total number of priority sites identified, species for which at least 1% of their respective populations occur in at least one of the priority sites, and IUCN red list status for globally threatened species (CR = Critically Endangered; EN = Endangered; VU = Vulnerable), as of 12 September 2023. See supplementary table 2 for the number of candidates assessed for each country.

Country	Number of priority sites identified	Species occurring in large congregations (≥ 1%) in priority sites
Bangladesh	8	16 species, including Spoon-billed Sandpiper *Calidris pygmaea* (CR), Indian Skimmer *Rynchops albicollis* (EN), Masked Finfoot *Heliopais personatus* (CR), and several anatid sp. (e.g., Ferruginous Duck *Aythya nyroca*, Common Shelduck *Tadorna tadorna*)
Cambodia	9	12 species, including Spot-billed Pelican *Pelecanus philippensis*, Sarus Crane *Grus antigone* (VU), Greater Adjutant *Leptotilos dubius* (EN), Masked Finfoot (CR), Asian Openbill *Anastomus oscitans*, Painted Stork *Mycteria leucocephala*, Black-headed Ibis *Threskiornis melanocephalus*, and Spotted Greenshank *Tringa guttifer* (EN)
PRC (coastal)	37	32 species including Spoon-billed Sandpiper (CR), Far Eastern Curlew *Numenius madagascariensis* (EN), Spotted Greenshank (EN), and Great Knot *Calidris tenuirostris* (EN)
PRC (inland)	23	17 species including Swan Goose *Anser cygnoid* (VU), Lesser White-fronted Goose *Anser erythropus* (VU), Baer’s Pochard *Aythya baeri* (CR), Siberian Crane *Leucogeranus leucogeranus* (CR), White-naped Crane *Grus vipio* (VU), Red-crowned Crane *Grus japonensis* (VU), Hooded Crane *Grus monacha* (VU), Oriental Stork *Ciconia boyciana* (EN), and Relict Gull *Larus relictus* (VU)
Indonesia	17	19 species including Spotted Greenshank (EN), Asian Dowitcher *Limnodromus semipalmatus*, Red Knot *Calidris canutus*, Bar-tailed Godwit *Limosa lapponica*, Black-tailed Godwit *Limosa limosa*, and Australian Pratincole *Stiltia isabella*
Lao PDR	3	Masked Finfoot (CR), Asian Openbill, and Little Ringed Plover *Charadrius dubius*
Malaysia	6	11 species including Spotted Greenshank (EN), Far Eastern Curlew *Numenius madagascariensis* (EN), Great Knot (EN), Lesser Sandplover *Charadrius mongolus*, Broad-billed Sandpiper *Calidris falcinellus*, and Chinese Egret *Egretta eulophotes* (VU)
Mongolia	11	44 species including Dalmatian Pelican *Pelecanus crispus*, Hooded Crane (VU), Swan Goose (VU), and Common Pochard *Aythya marina* (VU) – mostly at breeding localities
Philippines	12	27 species, including Great Knot (EN), Chinese Egret *Egretta eulophotes* (VU), Grey-tailed Tattler *Tringa brevipes* (NT), Red-necked Stint *Calidris ruficollis* (NT), Chinese Crested Tern *Thalasseus bernsteini* (CR) and several herons and anatid sp. (e.g., Tufted Duck *Aythya fuligula*, and Garganey *Spatula querquedula*)
Thailand	12	15 species including Spotted Greenshank (EN), Black-tailed Godwit, Great Knot (EN), and Sarus Crane *Grus antigone* (VU)
Viet Nam	9	11 species including Spotted Greenshank (EN), Black-faced Spoonbill *Platalea minor* (EN), Lesser Sandplover, Broad-billed Sandpiper *Calidris falcinellus*, Common Tern *Sterna hirundo*, Little Tern *Sternula albifrons*, and Painted Stork
Total	147	

**Table 4:** Priority sites in the 10 regional flyway initiative (RFI) countries.

Country	Number of RFI priority sites	Wetland type		Protection status		Human Development Index tier (2021/22)
		Coastal	Inland	Protected or partially protected	Unprotected	
Bangladesh	8	5	3	8	0	Medium
Cambodia	9	1	8	7	2	Medium
PRC	60	37	23	52	8	High
Indonesia	17	16	1	8	9	High
Lao PDR	3	0	3	2	1	Medium
Malaysia	6	6	0	2	4	Very high
Mongolia	11	0	11	10	1	High
Philippines	12	9	3	7	5	Medium
Thailand	12	9	3	8	4	Very high
Viet Nam	9	8	1	4	5	High
Total	147	91	56	108	39	

### Characteristics of the RFI priority sites

Of the 147 selected priority sites, the majority are coastal wetlands (62%), with inland wetlands accounting for only 38% of sites (χ² = 8.33, df = 1, *P* < 0.004). Similarly, the distribution of sites by protection status shows a skew, with 73% falling under protected or partially protected areas, compared to just 27% that are unprotected (χ² = 32.39, df = 1, *P* < 0.001). The difference in proportion of coastal and inland wetland sites between the two protection statuses (protected/partially protected vs. unprotected) is statistically significant as there were fewer unprotected inland wetland sites than expected and more unprotected coastal wetland sites than expected (χ² = 6.46, df = 1). Lastly, the distribution of sites across countries classified by HDI tiers further shows the imbalance — sites are predominantly situated in countries with high or very high human development (78% combined), with only 22% in medium human development countries (χ² = 72.53, df = 2, *P* < 0.001). Notably, none of the RFI countries belonged to the tier of low human development, highlighting a focus on regions with higher development indices.

## Discussion

### Selection of the RFI priority sites

The Regional Flyway Initiative aims to prioritise conservation and management interventions for the most important wetland sites for migratory species through a data-driven site selection process to assess internationally important wetlands in 10 Asian countries. In our analyses, we adopted site selection criteria in alignment with the quantitative criteria developed for three long-established, programmes and initiatives for wetland conservation in Asia: the Ramsar Convention on Wetlands, the Flyway Site Network of the EAAFP, and the BirdLife International’s civil-society led IBA Programme. The number of selected priority sites was more than expected, primarily because more inland sites were included on the list than originally planned. While the original focus of the RFI was on coastal wetland sites, two countries within the geographical scope of the initiative—Mongolia and Lao PDR—are landlocked. Based on recommendations from national experts, inland wetlands within the Yangtze and Yellow River basins, as well as in Northeast PRC, were also included. This expansion was due to the presence of waterbird species supported by these wetlands and the distinct economic and social benefits they offer, which differ from those associated with sites in the more prosperous coastal provinces of the PRC.

To interpret the analyses in each country, it is important to be aware of the variations in (i) the geographic distributions and abundance of migratory waterbird populations within the EAAF; (ii) the characteristics of the wetlands in different parts of the flyway; and (iii) the availability of data on the numbers of migratory waterbirds that occur at wetland sites, as well as differences in the geographic sizes and topography of the countries. National waterbird and wetland experts provided guidance on how to factor these into the selection of the priority sites. Caution should be exercised in making comparisons between countries because of variations in distribution of various waterbird species, the characteristics of the wetland sites, and the availability of data.

This study advances knowledge over the work of Jaensch^36^ conducted for the EAAFP in three key ways: first, the selection of priority sites was based on the most accurate and up-to-date waterbird count data available; second, the approach was bottom-up, involving consultations with local and national stakeholders who possess in-depth knowledge of the sites and their migratory waterbirds, including national experts and site managers; and lastly, the selection process took into account precise site boundaries, resulting in more accurate identification of the actual locations of the priority sites.

### Site-based approach to conservation

In this study, we focus on the site-based approach as a pivotal tool for biodiversity conservation, recognising its limitations and role within a broader context of conservation strategies^78^. The dispersed nature of migratory species renders the conventional site-based approach insufficient for their effective conservation^79^. For example, many waterbird species covered in this study may be highly dispersed during the breeding season but congregate in large flocks during the migration passage and non-breeding period, which is when they are in habitats that are most threatened in their entire distribution as a result of bottleneck, or are vulnerable to hunting pressure. Conservation of a set of important sites can be an effective approach for migratory species if sites prioritised are of high importance for the species, which can be judged based on high proportions of the whole population being present at the site. Traditionally, actions to conserve migratory taxa have been associated with a site-based approach, leveraging the concentration of these species at specific wetlands (e.g., Mehlman et al..^80^). However, our site-based approach should be considered one among multiple strategies in the conservation toolbox. Our findings provide for a framework of a threefold approach to migratory species conservation:

1. Highlighting species-specific strategies focused on globally threatened birds within a flyway (e.g., Black-faced Spoonbill, Spoon-billed Sandpiper). This can direct resources toward their conservation. While an individual species focus might not always yield cost-effectiveness (see Lloyd et al.^81^), it could guide conservation investments for sites of importance to many species due to the perceived importance of these species (see Fitzpatrick et al.^82^).
2. Selecting a strategic set of sites that are designed to generate tangible impacts on bird populations^83^ at the range-wide and continental level. The site-based approach remains the cornerstone of our project. However, the historical piecemeal implementation of this approach across East Asian-Australasian Flyway has limited its impact. Our targeted site-based network strategy marks a significant departure from previous efforts in Asia and holds promise for population-level changes^84^.
3. Emphasising the need for policy-driven strategies. These encompass interventions at broader scales, with agricultural policies and investments in landscape-altering platforms akin to the Farm Bill in the United States or the Structural Funds and The Birds Directive^85^ in the European Union. Such policies can address the conservation needs of migratory species that transcend site-specific boundaries (e.g., Great Lakes Commission^86^.

The synergy among these three strategies is essential for effective biodiversity conservation and challenges the notion that a single approach can suffice, emphasising the importance of an integrated conservation strategy. Despite the limitations of the site-based approach, it is vital to recognise its role within a larger ecological network. Species losses are driven by a wide range of factors, and while the site-based approach can mitigate local threats, it may not fully address global drivers of change such as climate change. To enhance the effectiveness of these strategies, incorporating human dimensions is crucial. Engaging stakeholders—from local inhabitants and wetland site managers to local and national government officials and conservation practitioners—can provide valuable insights into how people value, interact with, and impact natural resources^87^. Such engagement at local to regional levels can elucidate cultural norms, community values, and social dynamics, which are critical for building partnerships, fostering community-led conservation initiatives, and ensuring that interventions are culturally appropriate and socially sustainable.

Furthermore, extending the prioritisation framework to include human population demographics at continental to global scales can improve understanding of how human pressures influence conservation needs and opportunities across different regions. Recognising the constraints of limited budgets and resources, an effective approach must also consider logistical and financial feasibility—prioritising sites where conservation efforts can be most cost-effective and impactful. In addition, integrating considerations of ecosystem services—for example, wetlands that provide flood protection, or support local livelihoods through sustainable fisheries—can help align biodiversity conservation goals with human wellbeing. Such a holistic approach ensures that efforts are not only ecologically sound but also socially relevant and economically feasible, ultimately enhancing the resilience of both ecosystems and human communities.

### Limitations in knowledge and data on waterbird populations

Using data from diverse sources for our analyses, we acknowledge that there are considerable variations and uncertainties in the count data of shorebirds from surveyed sites across 10 countries. Some wetlands in the EAAF are regularly counted or surveyed, for example, many of the sites covered by the AWC, the China Coastal Waterbird Census and in the lower Yangtze basin in the PRC, meaning that relatively comprehensive data was available for some sites to be assessed using our RFI methodology. Few wetland sites in Asia are intensively monitored, for example, the Luannan-Zuidong coast in Hebei Province, Chongming Dongtan in Shanghai and Mai Po in Hong Kong, PRC and parts of the Inner Gulf of Thailand, but these are very much the exception. Other than a few well-known sites, many wetlands in Southeast Asia are even more sporadically monitored, and the majority of sites have been counted only occasionally, perhaps only once or twice in the past 10 years, if not less.

Mixing single counts with averages over multiple surveys can potentially lead to misleading inferences about waterbird population sizes and site importance. Single counts may reflect temporary or anomalous conditions, such as seasonal fluctuations, and may not accurately represent typical abundance levels. Conversely, averages derived from multiple counts can smooth out such variability but may still be influenced by infrequent or inconsistent sampling efforts. Regularly monitored sites tend to have higher maximum counts of waterbirds and longer lists of species that regularly exceed the relevant 1% population thresholds. The PC1 scores of many of the lesser-known sites will be perennially underestimated because of the incomplete data available on migratory waterbirds, but some of these sites will nevertheless have been selected as priority sites based on this limited data available. This variability and uncertainty could result in overestimating or underestimating the importance of certain sites, thereby affecting prioritisation decisions. This highlights the need for caution when interpreting these data and the importance of standardised, regular monitoring to improve accuracy. Sites with large areas of potential habitat for waterbirds but lacking count data, along with those that do not overlap with protected areas (see Supplementary Table 3), should be prioritised for future monitoring.

Potential biases in expert opinion were also a consideration in our prioritisation process. Experts might have personal interests or familiarity with specific sites, which could influence their assessments and introduce bias towards certain areas. However, to minimise these biases, the site prioritisation was conducted through a participatory process that followed a standardised protocol. The final outcomes were based on a consensus among all participating experts, ensuring that the prioritisation reflected a collective, evidence-based judgment.

Another limitation of our study is the lack of consideration for habitat quality of the selected sites. While experts were asked to evaluate the current degree of habitat disturbance, the ongoing habitat loss and existing threats were not factored into the scoring process. This omission might impact the prioritisation by overestimating the importance of sites that are currently facing significant degradation. Moreover, we did not account for future threats such as human development, which could further endanger the wetland habitats. Sites with high potential for future loss or degradation might merit a higher priority to pre-emptively address these looming threats. Including habitat quality and future risk assessments could provide a more comprehensive prioritisation, ensuring that sites at risk of significant deterioration receive the attention needed to safeguard them against anthropogenic pressures.

### Implications for policy development and management

The selection of priority sites reveals a notable discrepancy between the prevalence of coastal and inland wetlands in the list. Recognising these differences is vital because each wetland type demands tailored policy development and management strategies. Coastal wetlands, which are abundant among the prioritised sites within countries such as Indonesia and Viet Nam, possess attributes such as storm buffering capabilities that protect coastlines from erosion and tidal surges^88^. Consequently, policies must prioritise their conservation and restoration to strengthen coastal resilience. In addition, maintaining the crucial balance of salinity within these tidal wetlands is essential, necessitating targeted policies to counteract threats such as industrial pollution and agricultural runoff, excessive ground water extraction, and reduced flows of fresh water from rivers.

In contrast, the prominence of inland wetlands within some countries such as Cambodia highlights their role in flood regulation, absorbing excess water during heavy precipitation events^88^. Effective policies should acknowledge their importance in mitigating flood risks and restrict urban development in vulnerable regions. The provision of drinking water by inland wetlands further highlights the need for policies to curb nearby pollution sources.

A substantial number of selected priority sites already have some protection status, either as designated protected areas or with substantial overlap with such areas. Future investments in these protected sites and specific management actions (e.g., eradication of invasive alien species; mangrove replanting) that benefit requirements of migratory birds offer opportunities to enhance protection for migratory birds, support local livelihoods, and strengthen climate resilience. However, a strategic focus on unprotected sites – especially unprotected coastal ones – for future investments can effectively allocate resources where management efforts are needed. Such investments may encourage the designation of these sites in the future which will also help governments meet the 30 × 30 target of the Global Biodiversity Framework. Contextualising these and other important sites for waterbirds within the flyway and visualising them could be undertaken through expansion of the Critical Site Network Tool^89,90^, developed by Wetlands International and BirdLife International, to the EAAF.

Considering the socioeconomic context, many priority sites are in countries classified as having very high or high human development tiers. Therefore, this presents an avenue for strategic investment targeting sites in Bangladesh, Cambodia, the Lao PDR, and the Philippines, four countries in the medium human development tier. Such an approach would ensure the preservation of ecosystem services provided by these wetlands, consequently contributing to an improved overall standard of living for their beneficiaries. Balancing these considerations will be essential as the participating countries navigate the policy development and management and restoration for their vital wetlands.

### Recommendations and future actions

The Convention on Migratory Species (CMS) assessed that the conservation needs of migratory species can be best represented in the newly adopted Global Biodiversity Framework through stronger consideration for ecological connectivity through management of networks of critically important sites and habitats used by migratory birds^91^. The EAAFP has prioritised building a chain of internationally important sites to support migratory waterbirds since its establishment in 2006 although ecological connectivity across this network of sites have not been fully investigated with the exception of a handful of species. Also, satellite-tracking studies of migratory shorebirds have shown that species in the region used wetlands not previously known as important sites (e.g., Great Knot *Calidris tenuirostris*^92^. There is a risk that little known stopover sites might be lost with deleterious effects on migratory connectivity even before they are properly surveyed and studied, given that each species uses a different suite and network of wetland sites during the breeding period, northward and southward (passage) migrations and during the non-breeding (northern wintering) period. Recent studies have investigated how the concept of ecological connectivity can be applied to migratory species (e.g., Xu et al.^83,93^), but the data available to assess connectivity in the EAAF, is currently limited^37^ particularly in Southeast Asia.

**Fig. 3:**
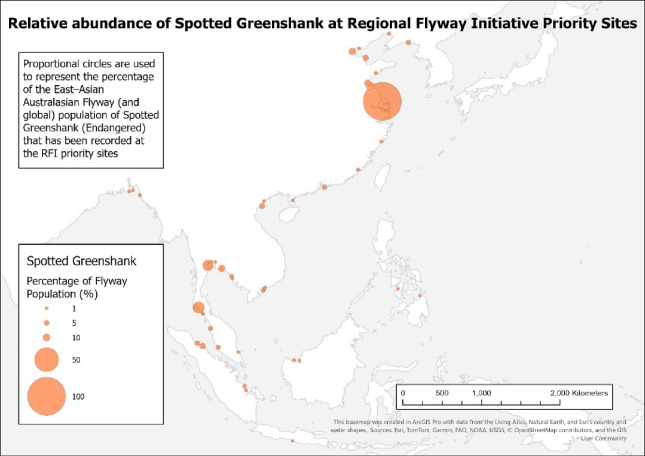
Representation of the Spotted Greenshank in Regional Flyway Initiative (RFI) priority sites across the East Asian-Australasian Flyway (EAAF). Proportional circles represent the percentage of the East-Asian Australasian Flyway (and global) population of Spotted Greenshank that has been recorded at the RFI priority sites (as of 12 September 2023).

Further investigations will need to consider whether globally threatened waterbirds are adequately represented in the prioritised set of RFI wetlands during the non-breeding period and the extent in which important sites for these species in the 10 RFI countries are missed or overlooked in our analyses. For example, the coverage afforded to the Endangered Spotted Greenshank *Tringa guttifer* by the 147 RFI priority sites (Fig. 3) has showed the (relative) irreplaceability of the wetland sites selected for that species.

Going forward, there is a need to ensure that the portfolios of wetland sites and landscapes are congruent with the priorities of each country in relation to national conservation strategies, development plans and various international obligations and resourcing opportunities. There is also a need to assess priority sites for their importance to various ecosystem services, in order to build a robust case for long-term management, including initiatives that will result from the implementation of RFI. The next steps include: (1) further consultation with government and civil society stakeholders to prioritise sites (for potential project development), delineate and agree on site boundaries, if needed; (2) assessment of ecosystem services provided by the priority sites; and (3) dissemination of RFI prioritisation findings to relevant stakeholders, and hence enhance ecological connectivity of the priority sites^94^. Through this comprehensive approach, the RFI seeks to safeguard the dynamic interplay between migratory waterbirds, wetlands ecosystems, and local communities within the EAAF.

## Conclusions

The site-based network approach, as explored in this study, is intended to contribute to the larger effort of biodiversity conservation with the focus on a continental-scale flyway in Asia. While not a panacea, when executed at a meaningful scale, it has the potential to stabilise and restore populations of some migratory bird species. The RFI highlights the need for a multipronged conservation strategy that encompasses species-focused interventions, site-based efforts, and policy-driven approaches. By embracing this holistic perspective, we aspire to catalyse lasting positive change in wetland conservation in the EAAF but recognising that important aspects – such as current and future threats, human dimensions, shorebird demographics and migratory connectivity – remained to be addressed.

## Supplementary Information

Below is the link to the electronic supplementary material.

Supplementary Material 1

Supplementary Material 2

## Data Availability

The data that support the findings of this study are available from Wetlands International but restrictions apply to the availability of these data, which were used under license for the current study, and so are not publicly available. Data are however available from MC, SW, DLY upon reasonable request and with permission of Wetlands International.
